# Using Eggshell Membrane as Nerve Guide Channels in Peripheral Nerve Regeneration

**Published:** 2013-08

**Authors:** Gholam Hossein Farjah, Behnam Heshmatian, Mojtaba Karimipour, Ali Saberi

**Affiliations:** 1 Neurophysiology Research Center, Department of Anatomy, Faculty of Medicine, Urmia University of Medical Sciences, Urmia, Iran; 2 Neurophysiology Research Center, Department of Physiology, Faculty of Medicine, Urmia University of Medical Sciences, Urmia, Iran; 3 Department of Anatomy, Faculty of Medicine, Urmia University of Medical Sciences, Urmia, Iran; 4 Faculty of Medicine, Urmia University of Medical Sciences, Urmia, Iran

**Keywords:** Eggshell membrane, Nerve regeneration, Rat, Sciatic nerve

## Abstract

***Objective(s):*** The aim of this study was to evaluate the final outcome of nerve regeneration across the eggsell membrane (ESM) tube conduit in comparison with autograft.

***Materials and Methods: ***Thirty adult male rats (250-300 g) were randomized into (1) ESM conduit, (2) autograft, and (3) sham surgery groups. The eggs submerged in 5% acetic acid. The decalcifying membranes were cut into four pieces, rotated over the teflon mandrel and dried at 37^°^C. The left sciatic nerve was surgically cut. A 10-mm nerve segment was cut and removed. In the ESM group, the proximal and distal cut ends of the sciatic nerve were telescoped into the nerve guides. In the autograft group, the 10 mm nerve segment was reversed and used as an autologous nerve graft. All animals were evaluated by sciatic functional index (SFI) and electrophysiology testing.

***Results:*** The improvement in SFI from the first to the last evalution in ESM and autograft groups were evaluated. On days 49 and 60 post-operation, the mean SFI of ESM group was significantly greater than the autograft group (*P*< 0.05). On day 90, the mean nerve conduction velocity (NCV) of ESM group was greater than autograft group, although the difference was not statistically significant (*P*> 0.05).

***Conclusion:*** These findings demonstrate that ESM effectively enhances nerve regeneration and promotes functional recovery in injured sciatic nerve of rat.

## Introduction

Peripheral nerves can be often damaged due to crush, compression, stretching, avulsion or division (1). Current gold standard for the clinical treatment of severe peripheral nerve damage involves using an autologous nerve to bridge the defect in the injured nerve (2, 3). This method has been shown to be effective, but has several disadvantages, including an extra incision for removal of a healthy sensory nerve ultimately resulting in a sensory deficit at the donor site (4, 5). Even under optimal operating conditions, suture repair and nerve grafting do not always result in return of function, and neuroma and scar tissue commonly occure (6). In an effort to overcome these limitations, the use of nerve guidance channels (NGCs) to bridge the gap between severed nerve ends is being extensively explored (2, 3). NGCs are either natural or synthetic tubular conduits that are used to bridge the gap between injured nerve stumps (3). Vast amounts of research are currently being pursued to engineer the ideal NGCs that can promote both sensory and motor functions (7).

The chicken eggshell and its membranes are an inexpensive and abundant waste material which exhibit interesting characteristics for many potential applications (8). The chicken eggshell comprises calcified shell and shell membranes including inner and outer membranes. These membranes retain albumen and prevent penetration of bacteria (9). The discovery of eggshell membrane (ESM) as a natural source of combined glucosamine, chondroitin and hyaluronic acid has prompted treatment for osteoarthritis (10). The ESM have a high content of bioactive components, as well as properties of moisture retention and biodegradability that have potential use for clinical, cosmetic, nutraceutical and nanotechnology applications (8).

ESM is suitable for the adherence of stromal cells (11), and a biological dressing for burn (12). Based on its composition, various applications and ease of processing, ESM may have a great potential in clinical practice such as wound dressing and tissue engineering scaffold (13). ESM is non-toxic (14) and biodegradable (15). It may have great potential for nerve guide in studies of axonal regeneration in peripheral nervous system (8). The hen eggshell membrane protects the fetus just as the human amniotic membrane does (11).

Despite the ESM availability and its use in traumatic lesions of various tissues, its effects on the peripheral nerves have not been determined.

The purpose of this experiment study is two-fold; first, to describe the method we have developed for preparing and manufacturing the ESM tube; second, to evaluate the final outcome of nerve regeneration, both functionally and electrophysiology, across the ESM tube conduit in comparison with autograft.

## Materials and Methods

 Thirty adult male Sprague-Dawely rats weighing 275 to 300 g, were randomized into three groups, including (1) ESM conduit (n=10), (2) nerve autograft (n=10), and (3) sham surgery (n=10). The experimental procedures were approved by the ethical committee of Urmia University of Medical Sciences. The left sciatic nerve was used as the experimental side and the right sciatic nerve as the control. 

***Preparation of ESM tube***

 The eggs in the experiments were commercially available hen eggs. Four fresh raw eggs were washed with water and methanol. The eggs were opened at their blunt ends, making holes with 2 to 3 cm diameter. The fluid contents were poured out. The remaining calcareous cups were rinsed inside and out with water and then submerged completely in 5% acetic acid for about 8 days. A glass ball was placed in each empty eggshell as an aid in keeping both the inner and outer surfaces fully submerged in the 5% acetic acid. As the decalcification was proceeding, the residual sacs of shell membrane were rinsed daily in tap water and the proteinaceous residue of decalcified shell was gently removed mechanically insofar as possible. The decalcifying membranes were then returned to fresh 5% acetic acid. The procedure was repeated daily during a 6 to 8-day period until the membranes were soft and completely free of brittle eggshell remnants. The sacs were then cut into four pieces (16). These films were immersed in phosphate buffered saline of pH 7.4 for a period of 30 min. The ESM film was taken out and rotated over the teflon mandrel manually under sterile conditions to achieve a longitudinal orientation. The teflon mandrel along with the so formed ESM tube was removed a washed exhaustively with double distilled water for a period of 3 h and dried at 37^° ^C for 24 hr in a sterile laminar flow hood. The ESM tubes were then individually packed and sterilized with ethylene oxide for 24 hr at room temperature (15). The Tubes were stored indefinitely in the refrigerator after sterilization, and remove a few as needed for each experiment.The ESM nerve guide measured 2 mm in inner diameter, 12 mm in length, and wall thickness of 0.6 mm. 

***Surgical procedure***

Under general anesthesia with intraperitoneal Ketamine (90 mg/kg) and Xylazine (10 mg/kg), and after routine preparation of the operative field (hair trimming, 20% iodine ethylic alcohol solution) the entire left sciatic nerve was exposed through a 3-4 cm long posterolateral longitudinal straight incision on the lateral side of the left thigh followed by a gluteal muscle splitting incision. The sciatic nerve was exposed and isolated from the adjacent tissues. The operation was terminated at this point in sham operated group. A 10-mm nerve segment was removed proximal to the tibial and peroneal nerve bifurcation, leaving a gap of approximately 10 mm due to retraction of the nerve stumps. In the ESM group, nerve conduit, both the proximal and distal cut ends of the sciatic nerve, were telescoped into the ends of the nerve guides and fixed with a single 10-0 nylon epineurial suture. Before inserting the distal stump, the guides were filled with physiologic normal saline in order to prevent trapping of air bubbles within their lumens. In the Autograft group, the 10-mm nerve segment, being removed from the sciatic nerve, was reversed and used as an autologous nerve graft. On both proximal and distal coaptation sites, single 10-0 nylon epineurial sutures were used. The muscle was closed with 4-0 Dexon sutures and the skin was stitched with 3-0 nylon. Surgery was performed under aseptic conditions using an operating microscope. Following the surgery, animals were housed in individual cages with food and water ad libitum and a cycle of 12 h light/12 hr dark.

***Functional tests***

One day prior to surgery and afterwards on the 7th, 21st, 35th, 49nd, 60th and 90th day post-operation, Indian ink was applied to the plantar surface of the hind feet to cover the entire anatomical regions. Each animal was placed in a walking pathway ending in a darkened cage. White paper cut to the appropriate dimensions was placed at the bottom of the track. The rats hind feet were dipped in a specific type of paint, and the animal was permitted to walk down the track, leaving its hind footprints on the paper. The animals were evaluated to obtain three footprint parameters including: the distance between the first and the fifth toes: toe spread (TS), the distance between the second and the fourth toes: intermediary toe spread (IT), and the distance between the tip of the third toe and the most posterior part of the foot in contact with the ground: print length (PL). Measurements of footprint parameters were taken from the normal (N) and the experimental (E) limbs. The footprints of both operated and unoperated limbs were used to calculate the Sciatic Functional Index (SFI) using the formula developed by Bain et al (17): 

SFI= -38.3 [(EPL-NPL)/NPL] + 109.5 [(ETS – NTS)/NTS] + 13.3 [(EIT – NIT)/NIT] – 8.8 

The above formula was derived empirically, with the assumption that all four variables were equally important. The percentage change in each of the four variables was averaged and a weighting factor of 2.2 was included to give an average of 100% deficit as a result of total nerve destruction. An SFI value of 0 was considered normal, whereas an SFI of -100 meant total impairment, such as what would result from a complete transaction of the sciatic nerve. 

**Figure 1:**
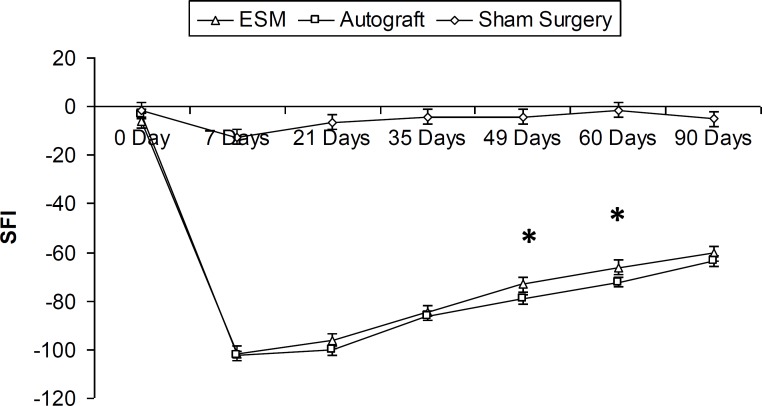
The sciatic functional index (SFI) before operation. After operation in ESM and autograft groups, SFI decreased to levels near – 100, representing complete loss of function (7 Days). * Difference between sham surgery group with the ESM and autograft groups (*P*<0.05, One-Way-ANOVA). Results are presented as mean ± SEM

***Electrophysiological study***

Ninty days after the operation, all animals were subjected to electrophysiological studies using Narco biosystem 320-3760 A trace 80 (USA). During the test, each rat’s body temperature was kept constant between 36.5- 37^°^C by mean of a temperature control unit (Narco, USA). Under anesthesia with intraperitoneal (IP) urethane (1 g/kg), the left sciatic nerve (the previousely-operated side) was re-exposed by incision of the previous surgical site at the mid-thigh level. Distal side of gastrocnemius muscle was detached from the attach endpoint of Achille^،^s tendon. A 1-0 thread was used to stitch the end of the muscle and tension converter. Single electrical pulses (at supramaximal intensity) were delivered via bipolar electrodes placed in turn at the proximal and distal trunks of the grafted nerve and recording electrodes were placed in the body of the gastrocnemius muscle. The length of nerve between stimulating and recording electrodes was measured. The physiologic parameters (Latency and amplitude of compound action potentials) were obtained and the nerve conduction velocity (NCV) was determined by stimulus latency and the distance between the two electrodes. The contralateral intact side was used for control parameters of electrophysiological and evaluation.

***Statistics***

Statistical analysis was done by a mixed-design (within and between the groups comparisons) ANOVAs were computed with 95% confidence intervals using the SPSS software (version 16.0). All data are presented as mean ± SEM and, p<0.05 was considered statistically signification. 

## Results

There were neither postoperative deaths nor clinical evidence of wound infections to be observed. The operations were well tolerated by animals and all wounds healed primarily. No clinical signs of infection, pain, or discomfort were observed during the regeneration period. 

The experimental parameters were analyzed from the day prior to surgery, and then on days 7th, 21st, 35th, 49nd, 60th and 90th post-operation. Preoperative SFI values (a day before surgery) did not differ between all experimental groups (-2.46 ± 3.71). The SFI was greatly decreased for both ESM and autograft groups 7 days after operation. The SFI improved from the first to the last evalution in ESM and autograft groups, since it increased from -101.6 ± 3.6 and -102.4 ± 2.55 during the 7th postoperative day to -60.46 ± 6.56 and -63.6 ± 3.96 during the 90th post-operation day, respectively.

On the days 49 and 60 post-operation, the mean SFI were -73.03 ± 5.19 , -66.09 ± 7.1 for the ESM group, and -77.77 ± 5.05 , -70.41 ± 3.72 for the Autograft group, respectively (*P*< 0.05). In addition, the ESM and autograft groups were statistically different from the control group (*P*< 0.05). There were no statistically significant differences between ESM and autograft SFI values 90 days post operation (Figure 1).

**Table 1:** Nerve conduction velosity (NCV) and maximum amplitude (AMP) comparisons in each group after operation

Group	NCV (m/S)	AMP (mV)
ESM	26.96 ± 4.72	4.75 ± 1.01
Autograft	25.01 ± 2.72	3.49 ± 1.68
Sham surgery	46.05 ± 3.78 ^*^	9.15 ± 1.28^*^
Control	49.08 ± 2.82^*^	9.42 ± 1.35^*^

* *P*<0.05, the difference between control / sham surgery and ESM /Autograft were significant. The difference between ESM and Autograft groups were not significant (*P*>0.05, One-Way-ANOVA). Results are presented as mean ± SEM.

NCV: nerve conduction velocity; AMP: amplitude; ESM: eggshell membrane

On the day 90th, the mean nerve conduction velocities (NCV) were 26.96 ± 4.72 and 25.01 ± 2.72 m/sec for the ESM and autograft groups, respectively. Although the NCV of the ESM group was faster than that of the autograft group, the difference was not statistically significant (*P*> 0.05). The mean amplitude (AMP) for the ESM and Autograft groups were 4.75 ± 1.01 mV and 3.49 ± 1.68 mV, respectively. The difference was not statistically significant (*P*>0.05). The results of the electrophysiological study are presented in Table 1.

## Discussion

The findings of this study demonstrated that ESM significantly enhances peripheral nerve regeneration in vivo. This is a pioneering study reporting the success of a nerve conduit made from ESM in a 10-mm sciatic nerve gap in rats. The quest for improvement in nerve regeneration is still one of the most challenging issues to surgeons and scientists (18). Although a definitive engineered alternative to autografts has yet to be identified, several promising methods are approaching the performance of autografts (19). 

In this study, chicken eggshell membrane (ESM) was shown to be a suitable material to be utilized in nerve regeneration. The ESM is a resorbable biomaterial for implant applications (20), a secondary surgical site is not needed to obtain ESM and there is no donor site morbidity. In addition, ESM can be obtained in large quantities, being inexpensive, sterilized with ethylene oxide and easily stored (15). Another advantage of using the ESM as a nerve guide conduit is that the degradation speed of this biological material can be controlled by manipulating the thickness of the tube (3).

The Chicken ESM contains glycoaaminoglycan (approximately 48% hyaluronic acid) (21). Hyaluronic acid has been shown to enhance peripheral nerve regeneration in vitro (22). High glycine content in all layers of the ESM suggests the presence of collagen in the avian eggshell protein matrix (23). The ESM contains collagen types I, V and X (24). The collagen tube has been shown to be very efficient as a nerve conduit (25). 

Our results demonstrate that both ESM and autograft repairs result in good regeneration. In our study, an observation period of 90 days was chosen, because the late complications (i.e., muscle atrophy and joint stiffness) have been found after 10 weeks (26). The most functional recovery occurred between the days 14 and 90 post-operation (27). The walking track analysis clearly demonstrated that there is a direct relationship between individual hind limb muscle function and print measurements (28). 

There are several reasons that make it difficult to compare our findings with studies of other groups concerning nerve grafting with collagen tubes: 1) Chemical composition and structure of the graft; 2) Length of the nerve gap; and 3) No published reports available for the usage of the ESM as NGCs.

Worthy of mention that, animals maintained a 50% deficit in nerve conduction velocity (NCV) in their operated leg on the 90th day post-operation. The decreased NCV is one of the consequences of the loss of large diameter fibers, a phenomenon reported in other traumatic nerve lesions (29). 

Despit many favorable functional findings for the ESM group, there were no significant differences in electrophysiology outcome for the ESM and Autograft groups.

There is a hypothesis that the walking track analysis is more comprehensive and reliable than histomorphometric methods in peripheral nerve repair studies (30-32). This study supports the idea that the combination of SFI with electrophysiological assessment is more comprehensive than electrophysiological method alone (33).

Electrophysiologic evalution was performed to study the restoration of contractile properties of reinnervated muscles. Nerve conduction velocity measures the fastest conducting nerve fibers, a measure that has been shown to be dependent on axon diameters, myelination and intermodal distance (34). A nerve may have a few fibers that conduct very well albeit a large number of remaining fibers are damaged. Hence, nerve conduction velocity may not evaluate total nerve function, although it will evaluate the fastest, possibly healthiest, fibers (32).

## Conclusion

Our findings demonstrate that the ESM effectively enhances nerve regeneration, with a mechanism still to be explained. However, further studies are needed to evaluate the efficiency of this ESM tube in larger gaps, and in association with substances that promote nerve regeneration, such as collagen, laminin, fibronectin, and various growth factors.
